# Fluid Dynamic Modeling to Support the Development of Flow-Based Hepatocyte Culture Systems for Metabolism Studies

**DOI:** 10.3389/fbioe.2016.00072

**Published:** 2016-09-30

**Authors:** Jenny M. Pedersen, Yoo-Sik Shim, Vaibhav Hans, Martin B. Phillips, Jeffrey M. Macdonald, Glenn Walker, Melvin E. Andersen, Harvey J. Clewell, Miyoung Yoon

**Affiliations:** ^1^Institute for Chemical Safety Sciences, The Hamner Institutes for Health Sciences, Research Triangle Park, NC, USA; ^2^ScitoVation, LLC, Research Triangle Park, NC, USA; ^3^Joint Department of Biomedical Engineering, University of North Carolina, Chapel Hill, NC, USA; ^4^Joint Department of Biomedical Engineering, North Carolina State University, Raleigh, NC, USA

**Keywords:** computational fluid dynamics, fluid dynamic modeling, hepatocyte culture, metabolism, QuasiVivo, RealBio

## Abstract

Accurate prediction of metabolism is a significant outstanding challenge in toxicology. The best predictions are based on experimental data from *in vitro* systems using primary hepatocytes. The predictivity of the primary hepatocyte-based culture systems, however, is still limited due to well-known phenotypic instability and rapid decline of metabolic competence within a few hours. Dynamic flow bioreactors for three-dimensional cell cultures are thought to be better at recapitulating tissue microenvironments and show potential to improve *in vivo* extrapolations of chemical or drug toxicity based on *in vitro* test results. These more physiologically relevant culture systems hold potential for extending metabolic competence of primary hepatocyte cultures as well. In this investigation, we used computational fluid dynamics to determine the optimal design of a flow-based hepatocyte culture system for evaluating chemical metabolism *in vitro*. The main design goals were (1) minimization of shear stress experienced by the cells to maximize viability, (2) rapid establishment of a uniform distribution of test compound in the chamber, and (3) delivery of sufficient oxygen to cells to support aerobic respiration. Two commercially available flow devices – RealBio^®^ and QuasiVivo^®^ (QV) – and a custom developed fluidized bed bioreactor were simulated, and turbulence, flow characteristics, test compound distribution, oxygen distribution, and cellular oxygen consumption were analyzed. Experimental results from the bioreactors were used to validate the simulation results. Our results indicate that maintaining adequate oxygen supply is the most important factor to the long-term viability of liver bioreactor cultures. Cell density and system flow patterns were the major determinants of local oxygen concentrations. The experimental results closely corresponded to the *in silico* predictions. Of the three bioreactors examined in this study, we were able to optimize the experimental conditions for long-term hepatocyte cell culture using the QV bioreactor. This system facilitated the use of low system volumes coupled with higher flow rates. This design supports cellular respiration by increasing oxygen concentrations in the vicinity of the cells and facilitates long-term kinetic studies of low clearance test compounds. These two goals were achieved while simultaneously keeping the shear stress experienced by the cells within acceptable limits.

## Introduction

There has been a coordinated, international call to increase the use of *in vitro* and *in silico* methods for hazard identification and risk assessment (Dix et al., 2007; Williams et al., 2009; Adeleye et al., 2015; De Boer et al., 2015). In response, scientists have developed advanced three-dimensional (3D) cell cultures with more physiologically relevant microstructures and environments that better match those in the intact organism (Marga et al., 2012; Kostadinova et al., 2013; Shulman and Nahmias, 2013; Ballard et al., 2015). Compared with traditional two-dimensional (2D) cultures, 3D cultures hold the potential to generate biological responses from chemical exposures that are more predictive of human *in vivo* responses (Zare-Mehrjardi et al., 2011; Pineda et al., 2013).

The liver plays an important role in the systemic clearance of xenobiotics. Currently, freshly isolated primary hepatocytes are regarded as the most relevant experimental system to study hepatic metabolism, as well as metabolism-mediated effects of chemicals and pharmaceuticals (Lecluyse and Alexandre, 2010). Freshly isolated primary hepatocytes express most of the proteins found in the human liver, including those involved in metabolism, membrane transport, and receptor-mediated processes (Li et al., 1995; Vildhede et al., 2015; Yang et al., 2015). However, one of the major drawbacks with primary hepatocytes is the rapid change in phenotype observed in culture. Several techniques have recently been developed to stabilize hepatic phenotype over longer periods, which may enhance the predictive power of primary cell culture for *in vivo* responses (Vinci et al., 2010; Ballard et al., 2015). These advanced cell culture models have shown their ability to better mimic physiological microenvironments in the human liver in healthy and diseased states (Chan et al., 2013; Ballard et al., 2015). Recently, a dynamic culture device with a 3D scaffold for cell attachment [RealBio^®^ (RB)] was tested for long-term primary hepatocyte culture (Choi et al., 2014). In this method, cultured primary hepatocyte suspensions are seeded into a scaffold where they form 3D structures. The cultures remained viable for over 7 weeks and maintained some of their liver-specific functionality, e.g., sustained albumin production (Choi et al., 2014). Other similar advances have also been observed with several other culture methods (Dash et al., 2013; Ukairo et al., 2013; Ballard et al., 2015).

There is a growing appreciation that the use of these advanced culture systems can be beneficial for a variety of endpoints within many fields of research (Breslin and O’Driscoll, 2016; Clevers, 2016; Leek et al., 2016). In toxicology, bioreactors have the potential to improve chemical and drug safety assessment as they are expected to better recapitulate biological responses to chemical exposures. The combination of exposure control and ease of sampling with more organotypic cell cultures also make bioreactors attractive for kinetics and metabolism investigations. The potential ability to reproduce exposure profiles of parent chemical and metabolites combinations within culture systems can also improve concentration-effect responses and *in vitro* to *in vivo* extrapolations.

Many of the bioreactor systems are flow-based culture systems, and computational fluid dynamics (CFD) modeling is well suited to optimize bioreactor design as it provides a tool to describe fluid dynamics in the engineered culture devices (Kang et al., 2013; Hsu et al., 2014). CFD modeling allows parameter values to be varied and the effects on the system to be characterized rapidly. This includes effects that are easier to describe mathematically than to measure experimentally. For instance, CFD modeling can generate detailed descriptions of fluid flow and nutrient transport within bioreactor systems. It can also be used to describe spatiotemporal distributions of a test compound or oxygen within the system. CFD and other computational methods have previously been applied to enhance our understanding of system parameters fundamental for cell responses within complex 3D culture environments (Kang et al., 2013; Hsu et al., 2014).

In this study, CFD modeling was used to assist the development of a flow-based hepatocyte culture system for long-term hepatocyte culture suitable for extended investigations of metabolic clearance or metabolite profile identification of low clearance compounds. Previous data indicate that the scaffold-based RB system supports long-term hepatocyte viability but not maintained metabolic capabilities. We hypothesize that increased cell–cell interactions fostered in other types of 3D culture conditions in combination with dynamic flow culture could improve the phenotypic stability. Two other bioreactors, QuasiVivo^®^ (QV) and fluidized bed (FB), were tested for this reason. They do not require a specialized scaffold but are compatible with some existing 3D methods for long-term hepatocyte culture, including alginate-encapsulated hepatocytes. CFD models were developed for the RB, QV, and FB bioreactors with the aim to identify a bioreactor suitable for alginate bead culture.

## Materials and Methods

### Materials

William’s E medium was purchased from Life Technologies (Grand Island, NY, USA). Trypan blue (TB) and 7-ethoxycoumarin were purchased from Sigma-Aldrich (St. Louis, MO, USA). RB bioreactors were purchased from RealBio^®^ Technology, Inc. (Kalamazoo, MI, USA). Quasi-Vivo^®^ bioreactors were provided by Kirkstall Ltd. (Rotherham, UK). The FB bioreactor was manufactured by Professor Jeffrey M. Macdonald and Vaibhav Hans, University of North Carolina (Chapel Hill, NC, USA).

### Bioreactor Design

**Figure 1:**
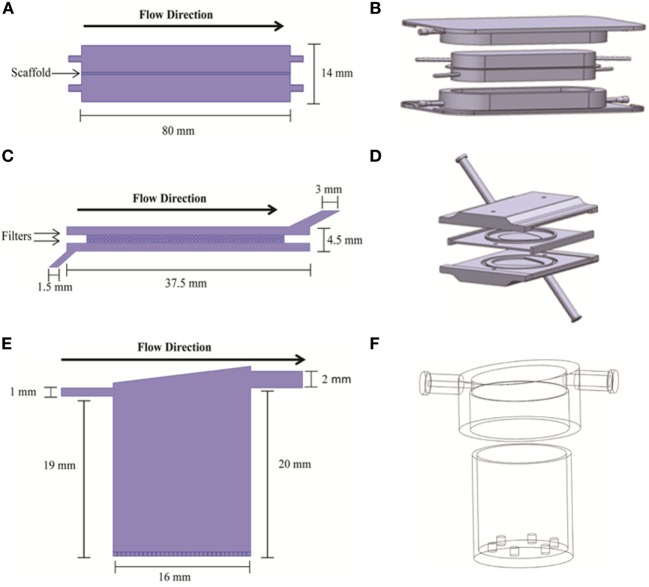
**2D (A,C,E) and 3D (B,D,F) geometry models of the bioreactors used in the simulations**. 2D models are from COMSOL; 3D models are from Solidworks. The three bioreactor types are RealBio **(A,B)**, fluidized bed **(C,D)**, and QuasiVivo **(E,F)**.

Three bioreactor systems (RB, FB, and QV) were selected for inclusion in the current study. Diagrams of these systems are shown in Figure 1.

The RB bioreactor (Figures 1A,B) is a scaffold-based system where hepatocyte suspensions are seeded and cultured on a 3D woven polycarbonate scaffold (Pfund et al., 2013). The 1-mm thick scaffold is maintained between two media compartments with dynamic flow. Both media compartments are in equilibrium with gas compartments through gas-permeable membranes (Figures 1A,B). All flows were modeled as concurrent, and all media inlets and outlets were of 1-mm internal diameter (ID). The total volume of the RB bioreactor is 7.0 mL.

The FB bioreactor (Figures 1C,D) was custom developed specifically for use with alginate-encapsulated primary hepatocytes. This bioreactor was machined from polysulfone and consists of three main parts: top and bottom pieces creating two chambers for media and a spacer piece wherein the cell compartment is located (Figures 1C,D). The FB bioreactor has a total volume of 4.8 mL. The cell compartment is separated from the media compartments with two polypropylene filters with 100-μm pore size. The media inlet was 1.5-mm ID, and the outlet was 3-mm ID.

The QV bioreactor (Figures 1E,F) has a single compartment with a single media inlet (1-mm ID) and outlet (2-mm ID) (Gordon et al., 2014). The volume of the chamber is 4.0 mL, and it can accommodate 2D or 3D cell cultures. 2D cultures are supported on a slide that rests on six small projections from the floor of the chamber, while alginate-based 3D cultures can be added to the chamber without the slide. The base of the bioreactor is made of Altuglas SG7 acrylic, while the lids are a styrene-based thermoplastic elastomer (TPE). The QV chamber has a polycylindrical shape with a slanted “roof” and offset outlet meant to promote mixing and reduce shear stress along the chamber bottom where the cells are located.

### Simulation Software

Bioreactor geometries were described using finite element modeling (FEM) algorithms as implemented in COMSOL Multiphysics 4.3 (The COMSOL Group, Stockholm, Sweden) and Solidworks 2013 (Dassault Systems, Waltham, MA, USA) (Figure 1). Most simulations were performed using COMSOL’s stationary solver that finds time-invariant solutions. Simulations of test compound distribution were performed using COMSOL’s time-dependent solver up to *t* = 60 min. The remaining simulations were performed using Solidworks.

### Fluid Dynamics Modeling

Flow velocity, turbulence, and shear stress profiles in each bioreactor were investigated using 2D and 3D CFD modeling. Fluid motion within the bioreactors was described by a Navier–Stokes equation assuming incompressible, isothermal Newtonian fluids. Equation 1 describes the momentum balance, while Eq. 2 describes continuity within incompressible fluids.

(1) $$\rho \frac{\text{∂u}}{\text{∂t}}-\mu \nabla^{2}u+\rho \left(u\cdot \nabla \right)u+\nabla P=F$$

(2) ∇·u=0

where ρ is density (kilograms per cubic meter), μ is viscosity (kilograms per meter per second), *P* is pressure (kilograms per meter per square second), *F* is volume force (kilograms per square meter per square second), *u* is the 3D velocity field (meter per second), and ▽ is del (aka nabla) operator, $\nabla ={\vec{e}}_{x}\frac{\partial }{\text{∂x}}+{\vec{e}}_{y}\frac{\partial }{\text{∂y}}+{\vec{e}}_{z}\frac{\partial }{\text{∂z}}$ for Cartesian coordinates (Buchwald, 2011). Model parameters used were viscosity = 10^−3^ kg/m/s, fluid density = 1000 kg/m^3^, temperature = 37°C, and no-slip boundary conditions. The volumetric flow rate was 0.5 mL/min (QV) or 1 mL/min (RB and FB). Outlets were modeled with a pressure of 0 kg/m/s^2^ with no viscous stress or environmental pressure.

Shear stress experienced by the cells was described with Eq. 3. The properties of cell culture media were assumed to be identical to those of water, the reference fluid.

(3) $$\tau_{\text{wall}}=\mu {\left(\frac{\text{∂u}}{\text{∂y}}\right)}_{y=0},\tau_{\text{wall}}=\tau_{\text{cells}}$$

where τ is shear stress (kilograms per meter per square second), μ is viscosity (kilograms per meter per second), *u* is flow velocity parallel to the wall (meter per second), and *y* is the distance to the wall (meters).

**Figure 2:**
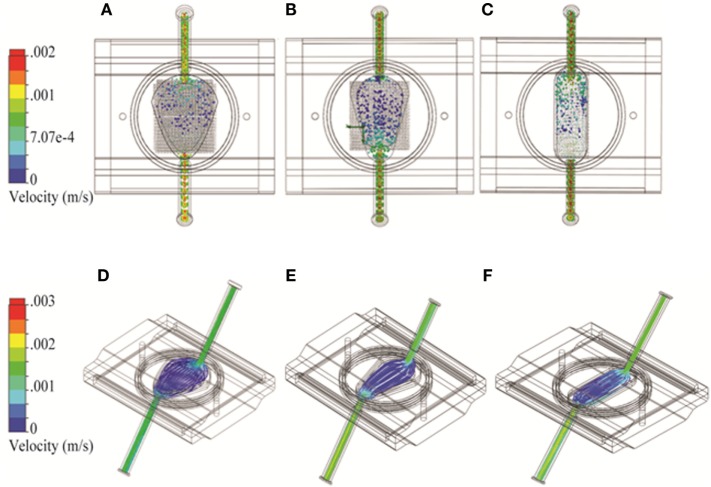
**Three variations on the fluidized bed (FB) bioreactor with different spatial dimensions using Solidworks**. **(A–C)** Flow particle study results and **(D–F)** flow trajectory study results shown from the top. Option 1: 27.3 mm maximum width **(A,D)**; Option 2: 22.2 mm maximum width **(B,E)**; and Option 3: 16.7 mm maximum width **(C,F)**.

Bioreactor fluid dynamic models were developed for 2D and 3D geometries. The pore size of the RB bioreactor scaffold was modeled as 100 μm. For the FB bioreactor, a porosity factor of 0.3 was used to simulate alginate beads in the cell compartment. Sensitivity analysis showed no significant differences in cell compartment flow for porosity values from 0.1 to 0.7. The filters on either side of the FB cell compartment were modeled as a 100-μm thick layer with evenly distributed pores of 100-μm diameter. To investigate the impact of the choice of spatial dimensions for the FB bioreactor, three variations of the width of the FB parabola were modeled: 27.3, 22.2, and 16.7 mm corresponding to the original (Figure 2A), narrow (Figure 2B), and oval (Figure 2C) design, respectively.

### Simulation of Small Molecule and Oxygen Distributions

The CFD models described in Section “Fluid Dynamics Modeling” were used to simulate the spatiotemporal distribution of oxygen and a representative small molecule in each bioreactor. Parameters describing these simulations are listed in Table 1.

**Table 1:** **Physical constants used in the CFD models to describe the mass transport of oxygen and a small molecule test compound in each bioreactor**.

Parameter name	Value	Reference
$D_{{\text{O}}_{\text{2}}}$, O_2_ diffusion coefficient in aqueous media	3 × 10^−9^ m^2^/s	Mazzei et al. (2010) and Buchwald (2011)
Cell-normalized O_2_ consumption rate in hepatocytes	0.4 nmol/s/10^6^ cells	Balis et al. (1999)
*k*_m_, Michaelis–Menten constant	5.6 mmHg	Foy et al. (1994) and Allen and Bhatia (2003)
*c*_cr_, critical O_2_ concentration	2.82 × 10^−3^ mol/m^3^	De Groot et al. (1988)
*C*_0_, O_2_ concentration at inlet	0.214 mol/m^3^	Mazzei et al. (2010)
$K_{{\text{O}}_{\text{2}}}$, Henry’s law constant	932.4 atm/mol/L	Mazzei et al. (2010)
*D*_test_, test compound diffusion coefficient	1 × 10^−8^ m^2^/s	Estimated
O_2_ solubility	0.2 mol/m^3^	USGS (2016)

#### Test Compound Distribution

In our simulations, the test compound enters the bioreactor through the inlet at a fixed concentration, as described in Section “Experimental Studies for Small Molecule Distribution.” Mass transport was by advection and diffusion, as described below (Eq. 4):

(4) $$\frac{\text{∂c}}{\text{∂t}}+\nabla \cdot \left(-D\nabla c\right)=R-u\cdot \nabla c$$

where *c* is the 3D chemical concentration (moles per cubic meter), *D* is the chemical-specific diffusion coefficient (square meter per second), *R* is the reaction rate (moles per cubic meter per second), and *u* is 3D velocity field (meter per second) (Buchwald, 2011). For the purpose of this study, metabolism was not included and *R* = 0 mol/m^3^/s for the test compound was used. The diffusion coefficient for a small molecule was set at 1 × 10^−8^ m^2^/s (Brody and Yager, 1997; Macdonald et al., 1999). A sensitivity analysis using a range of diffusion coefficients from 1 × 10^−6^ to 1 × 10^−10^ m^2^/s showed minimal influence of this parameter on the time it took to establish a uniform distribution of the test compound in the bioreactors.

#### Oxygen Distribution

Mass transport of oxygen was handled similarly to that of the test compound. Oxygen diffusion was described using Eq. 4 but using a diffusion coefficient of 3 × 10^−9^ m^2^/s (Buchwald, 2011). The concentration of oxygen in the media at *t* = 0 was assumed to be in equilibrium with the concentration of oxygen in air, as described by Henry’s law (Eq. 5).

(5) $$P_{{\text{O}}_{2}}=K_{{\text{O}}_{2}}\cdot C_{0}$$

where $K_{{\text{O}}_{2}}$ is the Henry’s law constant and $P_{{\text{O}}_{2}}$ is the partial pressure of oxygen in air. See Table 1 for a list of parameter values and references. The concentration was also used as the concentration of oxygen in the media entering the bioreactor, and the concentration of oxygen in the media at the air–liquid interface membranes in the RB and FB bioreactors.

In the presence of cells, local oxygen concentrations will depend on mass transport of oxygen in the bioreactor and the rate of oxygen consumption by the cells. The rate of oxygen consumption by the cells was assumed to follow Michaelis–Menten (MM) kinetics (Eq. 6). A step-down function was used to simulate the reduction in the rate of oxygen consumption as the local concentration of oxygen approaches the critical concentration for cell survival (2.8 μM) as previously described (De Groot et al., 1988). This approach has been shown to improve cellular oxygen consumption predictions at low oxygen concentrations.

(6) $$R=V_{\text{max}}\cdot \frac{c}{c+k_{\text{m}}}\cdot \delta \left(c>c_{\text{cr}}\right)$$

(7) $$\delta \left(c>c_{\text{cr}}\right)=\left\{\begin{array}{cc} 0\; & \;\;\;\text{for }c<-c_{\text{cr}}\\ 0.5+0.75\left(\frac{c}{c_{\text{cr}}}\right)-0.25{\left(\frac{c}{c_{\text{cr}}}\right)}^{3}\; & \;\;\;\text{for}-c_{\text{cr}}\leq c\leq c_{\text{cr}}\\ 1\; & \;\;\;\text{for }c>c_{\text{cr}} \end{array}\right.$$

where *V*_max_ is the maximal oxygen consumption rate (moles per cubic meter per second), *k*_m_ is the MM constant (moles per cubic meter), *c*_cr_ is the critical oxygen concentration (moles per cubic meter), and δ is the step-down function used to simulate the decline in oxygen consumption by the hepatocytes when local oxygen concentrations approach *c*_cr_ (Eq. 7). See Table 1 for a list of parameter values and references. A cell-normalized oxygen consumption rate of 0.4 nmol/s/10^6^ cells was used, along with a *k*_m_ value of 5.6 mmHg (Rotem et al., 1992; Balis et al., 1999). The total number of cells in the RB, FB, and QV bioreactors was estimated at 15, 70, and 1.5 million cells, respectively. *V*_max_ values were calculated by multiplying the cell-normalized oxygen consumption rate by the number of cells in the bioreactor and then dividing by the volume of the cell compartment. Cell numbers were estimated based on seeding density (RB) or the volume of alginate beads added to the bioreactor (FB and QV). Three bead diameters were considered: 250, 500, and 1000 μm. The alginate beads were assumed to be 50% (v/v) hepatocytes, and a hepatocyte diameter of 20 μm (Kuntz and Kuntz, 2008) was used to convert to total cell number. The FEM algorithm as applied in COMSOL was used to describe local oxygen concentrations within the beads. All oxygen transport simulations were run using the stationary solver.

#### Experimental Studies for Small Molecule Distribution

Two small molecules were used as test compounds, 7-ethoxycoumarin (7EC) and TB. Each was tested separately. The compound was added to the cell-free system using matched flow and other incubation conditions used for fluid dynamic modeling described above. The media was continuously recirculated through the system with a peristaltic pump. William’s E medium with 10 μM 7EC or 0.04% TB was flowed through each bioreactor in the absence of cells. The media leaving the reactor was sampled up to 60 min (RB) or 30 min (FB and QV) to determine compound distribution kinetics. Compound sample concentrations were determined by fluorescence (7EC, excitation at λ = 323 nm, emission at λ = 386 nm) or absorbance (TB, λ = 580 nm). Results were normalized to the maximum concentration that was measured in the time course.

## Results

### Optimizing Chamber Design in the FB Bioreactor

As the FB bioreactor was developed in-house, we were able to alter chamber geometry as needed. Three alternatives based on a shape with a parabolic cross-section were considered. The “original,” “narrow,” and “oval” designs had maximum widths of 27.3, 22.2, and 16.7 mm, respectively. The models were otherwise identical and were investigated for the impact of these design changes to flow pattern and turbulence. An uneven flow pattern with higher cross-flow through the cell compartment near the bioreactor outlet was observed in the original FB chamber (Figure 2A). With the narrow parabola design, the cross-flow pattern shifted to be stronger nearer the inlet (Figure 2B). The uneven flow pattern near the cell compartment was observed again near the outlet for the oval design (Figure 2C). No turbulence was observed in any of the FB chamber designs (Figures 2D–F).

### Bioreactor Flow Velocity, Turbulence, and Shear Stress

Computational fluid dynamics modeling simulated flow velocities, turbulence, and shear stress patterns within the RB, FB, and QV bioreactors. Both 2D and 3D models of velocity were examined. Turbulence and shear stress were analyzed using 3D models.

#### RB Bioreactor

**Figure 3:**
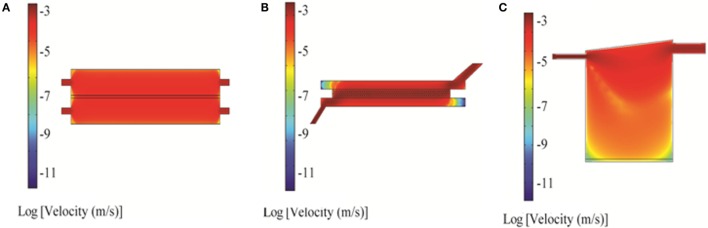
**2D simulation of flow velocity in the bioreactors using COMSOL: RealBio (A), fluidized bed (B), and QuasiVivo (C)**. The volumetric flow rate was 1 mL/min (RealBio and fluidized bed) or 0.5 mL/min (QuasiVivo).

**Figure 4:**
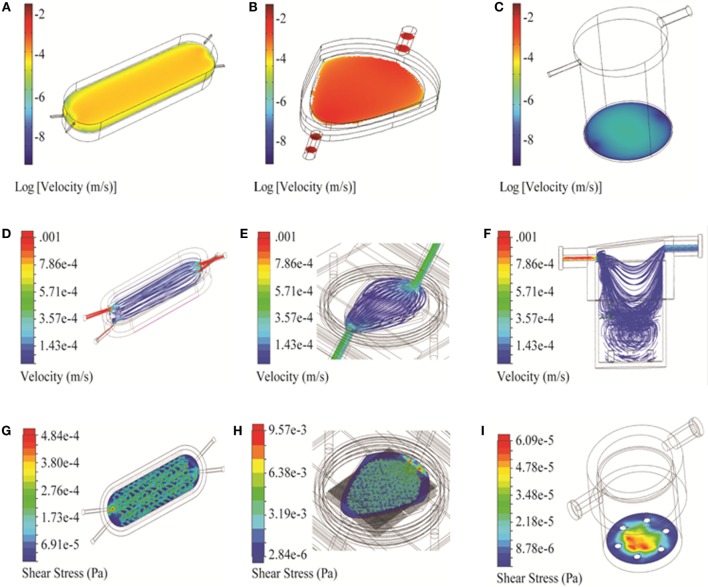
**3D simulation of flow velocity and shear stress in the bioreactors**. **(A–C)** Velocity profiles of bioreactors shown in the vicinity of the cell compartment/scaffold (COMSOL). **(D–F)** Flow trajectories, to identify turbulence within the chambers (Solidworks). **(G–I)** Shear stress profiles along the cell compartment/scaffold surface (Solidworks). Three bioreactors are shown: RealBio **(A,D,G)**, fluidized bed **(B,E,H)**, and QuasiVivo **(C,F,I)**.

Two-dimensional simulations of the RB bioreactor showed peak flow velocities of 2.9 × 10^−3^ m/s and 6.2 × 10^−4^ m/s at the chamber inlet and scaffold level, respectively (Figure 3A). 3D simulations showed peak flow velocity at the chamber inlet to be 0.0137 m/s, approximately five times higher than the 2D predictions. 3D simulations also showed velocities at the scaffold ranging from 1 to 8 × 10^−5^ m/s, depending on the position (Figure 4A). Flow was predicted to be laminar throughout the bioreactor, with turbulence possible only near the inlet and outlet where there are large constrictions or expansions (Figure 4D). Media cross-flow through the scaffold was observed between the two compartments, with predicted scaffold shear stress levels between 34.6 and 484 μPa (Figure 4G).

#### FB Bioreactor

Two-dimensional simulations of the FB bioreactor showed a peak inlet flow velocity of 8.02 × 10^−3^ m/s and cell compartment velocities ranging from 1 to 2 × 10^−3^ m/s (Figure 3B). 3D simulations showed a peak inlet velocity of 5.76 × 10^−3^ m/s and cell compartment flow velocities of 1–9 × 10^−4^ m/s (Figure 4B). The velocity in the cell compartment near the inlet was 9.8 × 10^−4^ m/s. No turbulence was observed in the FB bioreactor (Figure 4E). Cell compartment shear stress ranged from 1070 to 4260 μPa (Figure 4H).

#### QV Bioreactor

Two-dimensional simulations of the QV bioreactor showed a peak inlet flow velocity of 4.45 × 10^−3^ m/s and velocities near the alginate beads at the bottom of the chamber of 2 × 10^−7^ m/s (Figure 3C). 3D simulations showed a peak inlet velocity of 0.022 m/s, almost five times higher than the 2D predictions. The velocity near the alginate beads varied by position in the range of 3–15 × 10^−6^ m/s (Figure 4C). Some turbulence was observed in the QV bioreactor in the downward flow paths after the inlet, as well as at the periphery of the bottom of the chamber (Figure 4F). QV shear stress near the alginate beads varied by position in the range of 4–57 μPa (Figure 4I). These results are in good agreement with those previously reported for a variation of this bioreactor from the same manufacturer (Mazzei et al., 2010).

### Test Compound Mass Transport in the Bioreactors

#### CFD Modeling Results

**Figure 5:**
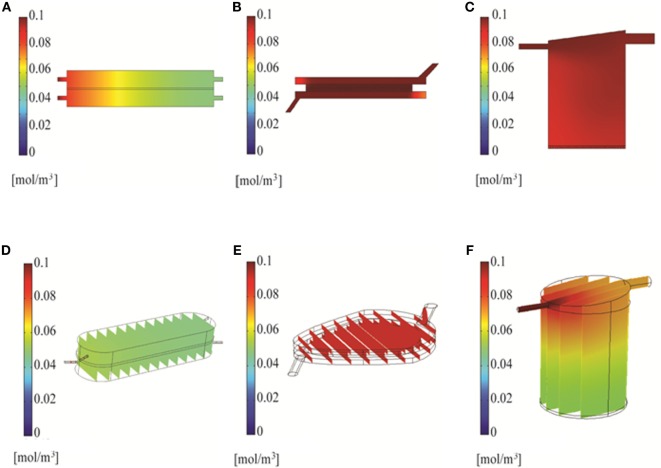
**Simulation of compound distribution in the bioreactor using Solidworks**. Chemical species transport simulated with 2D models at 6 min **(A–C)** or 3D models at 10 min **(D–F)**.

Two and three-dimensional CFD modeling was used to simulate mass transport of a representative small molecule in the three bioreactors. The 2D model predicted uniform compound distributions at 18, 6, and 6 min in the RB, FB, and QV bioreactors, respectively. Figures 5A–C show the concentration of a small molecule after 6 min. The corresponding 3D models predict it would take 40, 8, and 17.5 min for a small molecule to distribute uniformly in the RB, FB, and QV bioreactors, respectively. Figures 5D–F depict the concentration of a small molecule after 10 min. When compared, the FB bioreactor models resulted in similar predications using either 2D or 3D models. However, for the RB and QV bioreactors, the predicted time to equilibration was two to three times higher in the 3D predictions compared to the 2D.

#### Experimental Results

**Figure 6:**
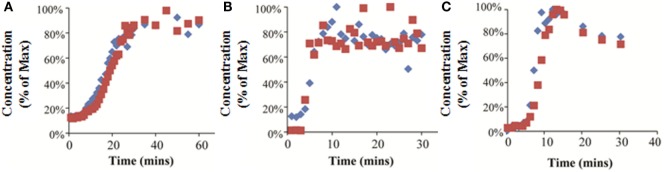
**Experimentally measured concentrations of 7-ethoxycoumarin (blue diamonds) or trypan blue (red squares) exiting the bioreactor, expressed as a percentage of the maximum detected concentration**. Results for RealBio **(A)**, fluidized bed **(B)**, and QuasiVivo **(C)**.

7-ethoxycoumarin and trypan blue were added to the media at the bioreactor inlet, and the media was sampled at the bioreactor outlet. The concentration at the outlet was normalized to the maximum concentration measured. The results of this experiment show that it took 25 min (RB), 8 min (FB), and 10 min (QV) for the outlet concentration to reach equilibrium (Figures 6A–C). In the QV bioreactor, the outlet concentration rose rapidly to a maximum and then began falling again (Figure 6C). This result is consistent with equilibration in a recirculating system where the flow initially carries the chemical through the bioreactor in a current along the top of the chamber, followed by slower back-mixing inside the chamber causing a drop in the outlet concentration over time. In this scenario, using the initial peak would underestimate the time to system equilibration.

For the FB bioreactor, both 2D and 3D CFD predictions were found to be in agreement with the experimental data. For the RB bioreactor, 2D predictions resulted in a 28% underestimation of the time to equilibration, while the corresponding 3D prediction resulted in a 60% overestimation. A similar pattern was observed for the QV bioreactor, and time to equilibration was underestimated by 40% by the 2D predictions and overestimated by 75% by the 3D predictions. In general, the 2D models averaged at a 1.6-fold underestimation of the time to equilibration, while the 3D models resulted in an average of 1.4-fold overestimation of the time to equilibration.

### Oxygen Distribution in the Bioreactors

**Figure 7:**
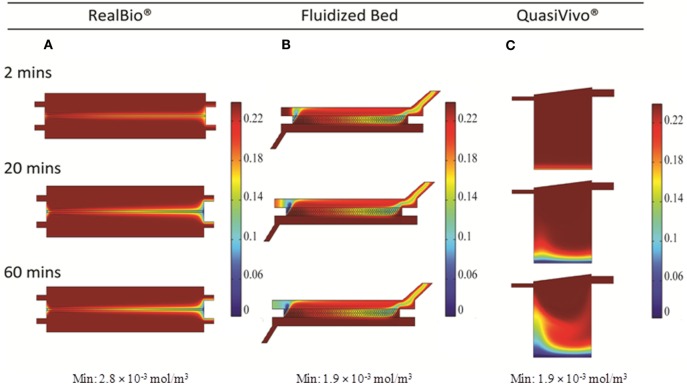
**2D simulation of oxygen concentration in the bioreactors using COMSOL**. Results for RealBio **(A)**, fluidized bed **(B)**, and QuasiVivo **(C)**.

**Figure 8:**
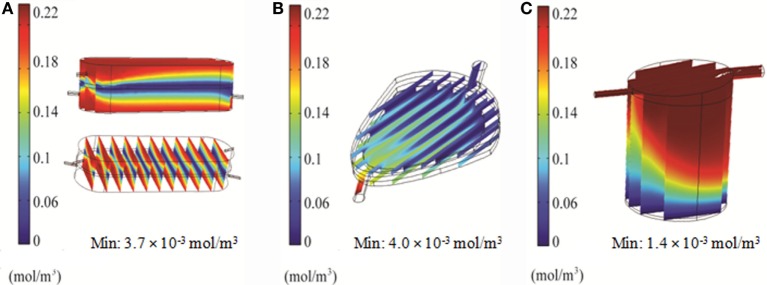
**3D simulation of oxygen concentration in the bioreactors using COMSOL**. Results for RealBio **(A)**, fluidized bed **(B)**, and QuasiVivo **(C)**.

Distribution of oxygen within the RB, FB, and QV bioreactors was simulated using 2D and 3D models. For the RB bioreactor, the 2D model predicted oxygen levels of 160 μM, well above the critical oxygen concentration, for the majority of the scaffold over the first hour in culture (Figure 7A). Using the 3D CFD model, oxygen concentrations down to 3.7 μM, less than twice as high as the critical oxygen concentration of 2.8 μM, were predicted closer to the chamber outlet and for parts of the center of the scaffold (Figure 8A).

In the FB bioreactor, 2D simulations indicate an average oxygen concentration of 130 μM in the area of the cell compartment after 60 min, with a moderate gradient ranging from 220 μM near the inlet to 100 μM at the outlet (Figure 7B). A low-oxygen area immediately above the inlet had a local velocity and oxygen level of 10^−9^ m/s and 1.9 μM, respectively (Figures 3B and 7B). However, the 3D simulation predicted a steady-state oxygen concentration of 4 μM throughout a majority of the cell compartment (Figure 8B). A higher oxygen of 12 μM was predicted around the inlet.

For QV, the 2D simulation results in oxygen levels above 200 μM for a majority of the cell compartment. An area with oxygen concentrations down to 7.5 μM in the area below the inlet was observed after 60 min (Figure 7C). The corresponding 3D simulation resulted in local oxygen concentrations as low as 1.4 μM at the cell level of the bioreactor (Figure 8C).

### Optimization of Flow Rate and Oxygen Partial Pressure in the QV Bioreactor

**Figure 9:**
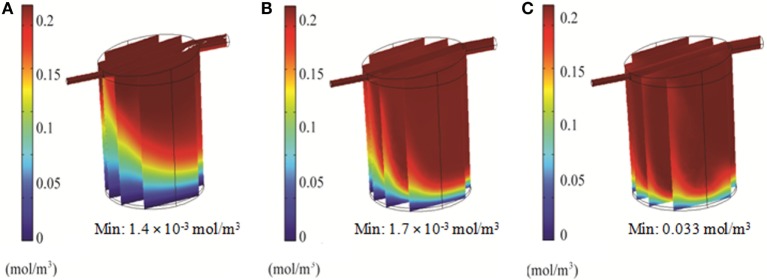
**3D simulation of oxygen concentration in the QuasiVivo for different flow rates using COMSOL**. Results for 0.5 mL/min **(A)**, 1.5 mL/min **(B)**, and 3 mL/min **(C)**.

**Figure 10:**
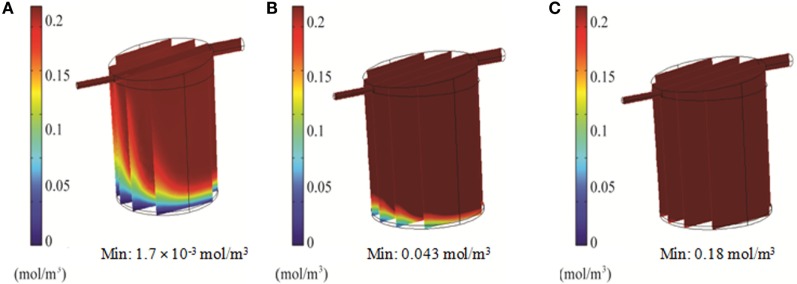
**3D simulation of oxygen concentration in the QuasiVivo for different oxygen percentages in the gas phase at a flow rate of 1.5 mL/min (COMSOL)**. Results for 21% O_2_ **(A)**, 35% O_2_ **(B)**, and 50% O_2_ **(C)**.

Simulations of the QV bioreactor showed favorable results for compound distribution and shear stress levels close to the alginate beads. However, predicted oxygen levels were too low to support the alginate-encapsulated 3D hepatocyte culture. Different media flow rates and external oxygen concentrations were therefore modeled to optimize the QV system for applications involving alginate-encapsulated 3D hepatocyte culture. As presented in Section “Oxygen Distribution in the Bioreactors,” the local oxygen concentration at the bottom of the QV bioreactor was predicted to be 1.4 μM at a flow rate of 0.5 mL/min. This local oxygen concentration increased to 1.7 and 33 μM at flow rates of 1.5 and 3 mL/min, respectively (Figure 9). Variations in local oxygen concentrations at the bottom of the QV bioreactor were investigated at the atmospheric level of 21% oxygen, as well as at 35 and 50% oxygen. When modeled using a 1.5 mL/min initial flow rate, the local oxygen concentration increased from 1.7 μM to 43 and 180 μM at 35 and 50%, respectively (Figure 10). The results suggest that increased flow rate and higher external oxygen concentration would be beneficial to increase QV applicability for alginate-encapsulated 3D culture of primary hepatocytes.

### Oxygen Distribution within Alginate Beads

Local oxygen concentrations within alginate and hepatocyte beads were investigated for 250, 500, and 1000 μm diameter beads using 2D and 3D simulations. The initial media concentration was set to 214 μM, as calculated using Eq. 5.

**Figure 11:**
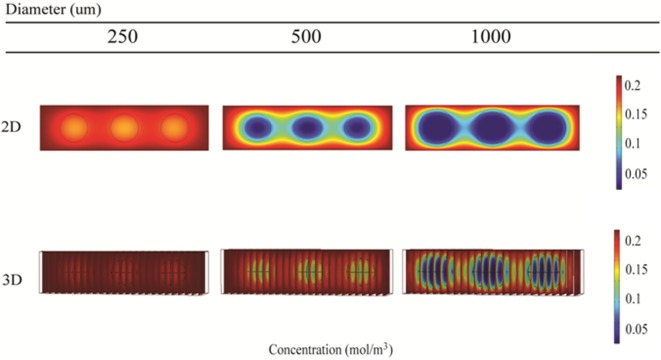
**Simulation of oxygen concentration inside beads of alginate-encapsulated hepatocytes using COMSOL**. 2D model results (top row) and 3D model results (bottom row) for the RealBio (left column), fluidized bed (center column), and QuasiVivo (right column) bioreactors.

The 2D simulations show steady-state oxygen concentrations at the center of the beads of 175, 25, and 4 μM for bead diameters of 250, 500, and 1000 μm, respectively (Figure 11, top row). All oxygen concentrations were predicted to be above the critical oxygen concentration of 2.8 μM, although by less than a factor of 2 for the 1000 μm beads. Corresponding 3D simulations resulted in steady-state oxygen concentration at the center of the beads of 200, 150, and 6 μM for bead diameters of 250, 500, and 1000 μm, respectively (Figure 11, bottom row).

## Discussion

Over the past decade, there has been an increase in the use of flow-based, long-term culture systems (i.e., “bioreactors”) for many purposes, including the investigation of organ function and cell metabolism. Several hepatic bioreactor systems have been developed with the aim to recapitulate parts of the complex *in vivo* microenvironment to better maintain the *in vivo* hepatocyte phenotype over time in culture. Typical parts of this microenvironment include extracellular factors, such as cell–cell interactions, cell–matrix interactions, chemical microenvironment, or physical parameters, such as shear stress (Kuntz and Kuntz, 2008; Lecluyse et al., 2012). Examples of advanced models include scaffold-based 3D cultures, spheroid cultures, or alginate-supported 3D culture. Several of these culture methods maintain hepatocyte morphology and viability over many weeks and previous results indicate that the inclusion of dynamic media flow extends the primary hepatocyte viability even further (Dash et al., 2013; Ukairo et al., 2013; Choi et al., 2014). With the expansion of the use of flow-based systems, CFD modeling has been an important tool to describe internal bioreactor parameters. CFD modeling enables rapid assessment of bioreactor parameters such as internal flow velocity, shear stress, and oxygen concentration that are difficult to determine experimentally. In the present study, CFD modeling was used in three different liver bioreactors as a long-term culture system for primary hepatocytes on 3D scaffolds or in alginate-encapsulated 3D beads. Bioreactor and operational parameters were optimized for use in chemical and drug safety assessments, more specifically for studying xenobiotic metabolism. It should be noted that all of the bioreactors investigated here were designed and optimized by their inventors for purposes other than long-term hepatocyte culture. Our goal was to identify design criteria for optimizing the bioreactors for assessing metabolism and to use computational modeling to explore the advantages and disadvantages of each system when used for our purposes.

An initial exploration was conducted to examine the effect of altering the spatial dimensions of the FB bioreactor to see if improvements could be made to the existing system. Examination of the results from the original system (Figures 2A,D) versus the two alternative systems (Figures 2B,E,F) showed no major improvements. Because no advantage could be observed, the original design was carried forward for future modeling studies.

Two- and three-dimensional models describing the geometry of the three bioreactors were built using two software platforms, COMSOL and Solidworks (Figure 1). Velocity fields were generated using these models to better understand how fluid moves through each system. The RB and FB bioreactors both had simple velocity fields, with fluid moving from inlet to outlet past and through the hepatocyte layer (Figures 4D–E). The RB bioreactor contains a scaffold where hepatocytes are seeded directly, flanked on both sides by two media compartments that are in communication with reservoirs of air *via* gas-permeable membranes to facilitate greater media oxygenation. Each media compartment possesses its own inlet and outlet, but they are angled so as to encourage a small amount of cross-flow through the scaffold. The FB bioreactor has one inlet and one outlet and is seeded with alginate supported 3D cell cultures in the cell compartment situated between the inlet and outlet. Media entering the bioreactors must flow through the alginate beads before exiting from the opposite side of the bioreactor. In both cases, the flow is almost entirely unidirectional.

In this study, the QV bioreactor is used for the culture of alginate-encapsulated 3D hepatocytes, just as the FB bioreactor. The outlet is offset from the inlet resulting in the media flow tangential to the cell compartment at the bottom of the chamber. A substantial portion of the flow establishes a circulatory pattern with subsequent mixing in the lower half of the chamber (Figure 4F). Unlike the RB or FB bioreactors, the QV system had areas with negligible flow (Figures 4D–F).

Flow regime is another factor to be considered during the optimization process. Turbulent flow can affect local shear stress as well as introduce a chaotic element into the modeling process. No turbulence was predicted for the FB bioreactor and was minimal for the RB and QV bioreactors at the volumetric flow rates under consideration. As such, it was not a major consideration when choosing between the three systems.

Shear stress experienced in the vicinity of the cells was also modeled (Figures 4G–I). A low level of shear stress provides a better representation of the *in vivo* microenvironment and may improve hepatocyte viability and phenotype stability (Dash et al., 2013; Choi et al., 2014). Conversely, too much shear stress will introduce cell stress that will damage or even kill the cells. The QV bioreactor had the lowest shear stress, followed by the RB bioreactor, with the FB bioreactor having the highest levels.

Accurate kinetic modeling requires a high degree of certainty regarding both the magnitude and the timing of exposure. In the context of this study, it means that a test compound added to the system should, in the ideal case, rapidly establish a uniform concentration throughout the system. Through CFD modeling (Figures 5D–F) and experiment (Figure 6), it was determined that the FB and QV bioreactors both achieved this goal within the first 10 min after dosing. The RB bioreactor took more than twice as long to establish a uniform concentration.

In order to support cellular respiration, sufficient oxygen concentrations are required. CFD modeling showed that both the RB and FB bioreactors had a minimal oxygen concentration above the critical level throughout (Figures 8A,B). The lowest predicted oxygen concentrations for the RB were less than twice the critical oxygen concentration and were observed near the outlet of the chamber and along the center of the scaffold were the media flow was less. In our experiments with the RB system, a lower density of cells was observed in the central region and near the outlet (Figure S1 in Supplementary Material). These areas were predicted to have local oxygen concentrations close to the critical oxygen concentration and almost a complete lack of shear stress, which could have impeded hepatocyte viability. In the QV bioreactor, the oxygen concentration dropped below this level, to 1.4 μM (Figure 8C). However, by increasing the flow rate to 3 mL/min (Figure 9C), or by increasing the flow rate to 1.5 mL/min and supplementing additional oxygen gas to the system above 35% (Figures 10B,C), the minimum oxygen concentration was brought above the critical level. Even in the presence of sufficient oxygen in the surrounding media, diffusion and consumption of oxygen within the alginate beads themselves could lead to insufficient oxygen levels near their centers. Modeling of the beads (Figure 11) showed that a diameter ranging from 250 to 500 μm is preferable. Cell viability of alginate supported 3D cultures of rat hepatocytes was maintained through day 31 of culture in the lab (Figure S2 in Supplementary Material).

The main design parameters under consideration were (1) minimizing shear stress on the cells/alginate beads, (2) minimizing the time to establish a uniform distribution of test compound in the bioreactor, and (3) maintaining oxygen concentrations above the critical level. Taken together, the QV system was selected because it showed the lowest shear stress of any system under consideration, a short time scale for test compound distribution, and sufficient oxygen availability at higher flow rates. The low shear stress was attributable to the placement of the cells at the bottom of the bioreactor, away from the high velocity current at the top of the chamber. The rapid distribution of test compound was due to the flow pattern in the chamber, which promoted mixing down to the bottom without high velocities that could cause shear stress on the alginate beads.

In conclusion, CFD modeling was successfully used to analyze the applicability of three different bioreactors for extended hepatocyte culture, with the potential for improved phenotypic stability. The results show that CFD modeling correctly describes and improves rapid assessments and optimizations of bioreactor design as well as the identification of suitable systems for specific applications. *In vitro* long-term chemical metabolism and kinetic investigations in the QV bioreactor are ongoing.

## Author Contributions

JP designed experiments, acquired the in-lab experimental data, interpreted the simulation results, and wrote the manuscript. Y-SS ran the computational models and assisted with the in-lab experiments. VH designed the fluidized bed bioreactor. MP, JM, GW, MA, HC, and MY contributed to study design, interpretation of the simulation data, and revision of the manuscript.

## Conflict of Interest Statement

The authors declare that the research was conducted in the absence of any commercial or financial relationships that could be construed as a potential conflict of interest.
