# Supplementary material for: Fluid Dynamic Modeling to Support the Development of Flow-Based Hepatocyte Culture Systems for Metabolism Studies
Source: Front Bioeng Biotechnol. 2016 Sep 30;4:72. doi: 10.3389/fbioe.2016.00072 (PMC5044513; doi:10.3389/fbioe.2016.00072)
Supplement: Supplementary file 1 [file Presentation_1.PDF]

Supplementary Material

Fluid Dynamic Modeling to Support the Development of Flow-based Hepatocyte Culture Systems for Metabolism Studies

Jenny M. Pedersen, Yoo-Sik Shim, Vaibhav Hans, Martin B. Phillips, Jeffrey M. Macdonald, Glenn Walker, Melvin E. Andersen, Harvey J. Clewell III, and Miyoung Yoon*

*** Correspondence:** Corresponding Author: myoon@scitovation.com

1 Supplementary material and methods

1.1 Material

Dulbecco's modified Eagle's medium (DMEM) and fetal bovine serum were purchased from Life Technologies (Grand Island, NY, USA). Dexamethasone, L-glutamine, penicillin, streptomycin, insulin, transferrin, selenium, and alginate were purchased from Sigma-Aldrich (St. Louis, MO, USA). Hepatocyte maintenance medium (HMM) was obtained from Lonza (Basel, Switzerland). Propidium iodide and acridine orange were purchased from Nexcelom Bioscience (Lawrence, MA, USA).

1.2 Hepatocyte isolation and culture

Primary rat hepatocytes were isolated with a two-step liver perfusion technique using a previously published method (Lecluyse and Alexandre, 2010). In the RB bioreactor, primary hepatocytes with viability > 75% were seeded on the gelatin coated scaffold at a density of 15×10^6 cells per bioreactor and initially maintained at 37 °C and 5% CO_2 in DMEM supplemented with 5% (v/v) fetal bovine serum, 4 µg/ml insulin, 1 µM dexamethasone, 4 mM L-glutamine, 100 U/ml penicillin and 100 µg/ml streptomycin. Cells were allowed to attach to the scaffold for 24 hours, after which the medium was exchanged for HMM supplemented with insulin 10 µg/ml, transferrin 5.5 µg/ml and selenium 5 ng/ml, 0.1 µM dexamethasone, 100 U/ml penicillin and 100 µg/ml streptomycin.

1.3 Preparation of alginate-supported 3D hepatocyte cultures

Alginate-encapsulated primary hepatocytes were prepared from freshly isolated rat hepatocytes (QPS, RTP, NC) using a previously published method (Chandrasekaran et al., 2006). The beads were maintained at 37 °C and 5% CO_2 in HMM supplemented with insulin 10 µg/ml, transferrin 5.5 µg/ml and selenium 5 ng/ml, 0.1 µM dexamethasone, 100 U/ml penicillin and 100 µg/ml streptomycin. Alginate-encapsulated hepatocytes were cultured in the FB and QV bioreactors in an incubator set at 37 °C and 5% CO_2. The FB bioreactor was placed on an orbital shaker operating at 100 rpm (VWR 3500 Advanced Digital Orbital Shaker Mixer, Radnor, PA, USA).

1.4 Determination of cell viability

Cell viability was determined based on the concentration of lactate in the media (RB) or on a qualitative live-dead cell imaging analysis of the alginate-encapsulated hepatocytes (FB and QV). Lactate concentration was measured using a Lactate Plus analyzer (Nova Biomedical, Waltham, MA). For viability imaging, the alginate-encapsulated hepatocytes were stained with acridine orange and propidium iodide that bind to live or dead cells, respectively. The image of the beads taken at Day 31 shows that the encapsulated hepatocytes remain viable over this time period (Supplementary Figure S1).

2 Supplementary Figures

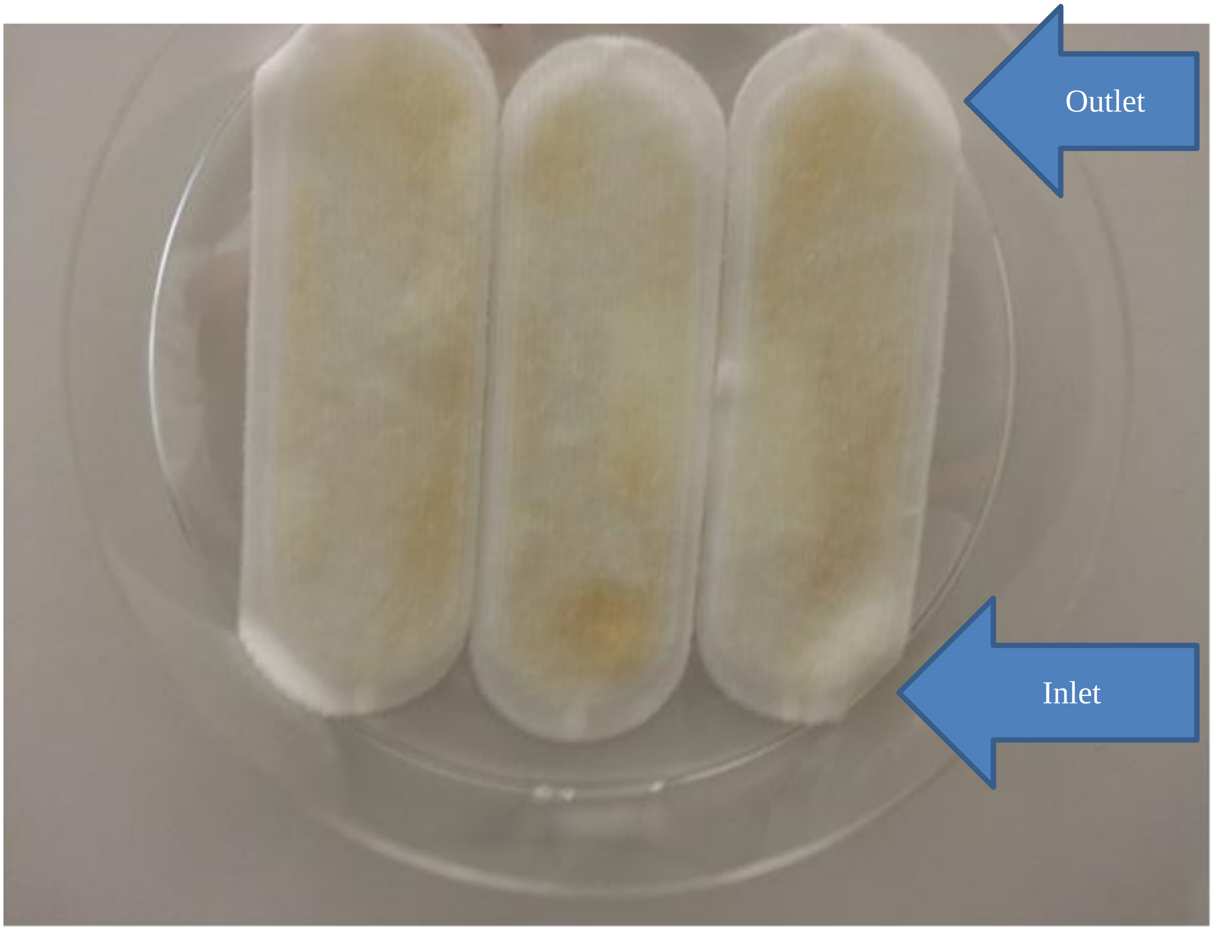

Supplementary Figure S1. Hepatocyte attachment to the scaffolds is better in areas of higher oxygen levels, close to the inlets and along the scaffold edges of the RealBio® system. The beige stains on the scaffold are attached hepatocytes at culture day 14. Note a large chamber-to-chamber variation.

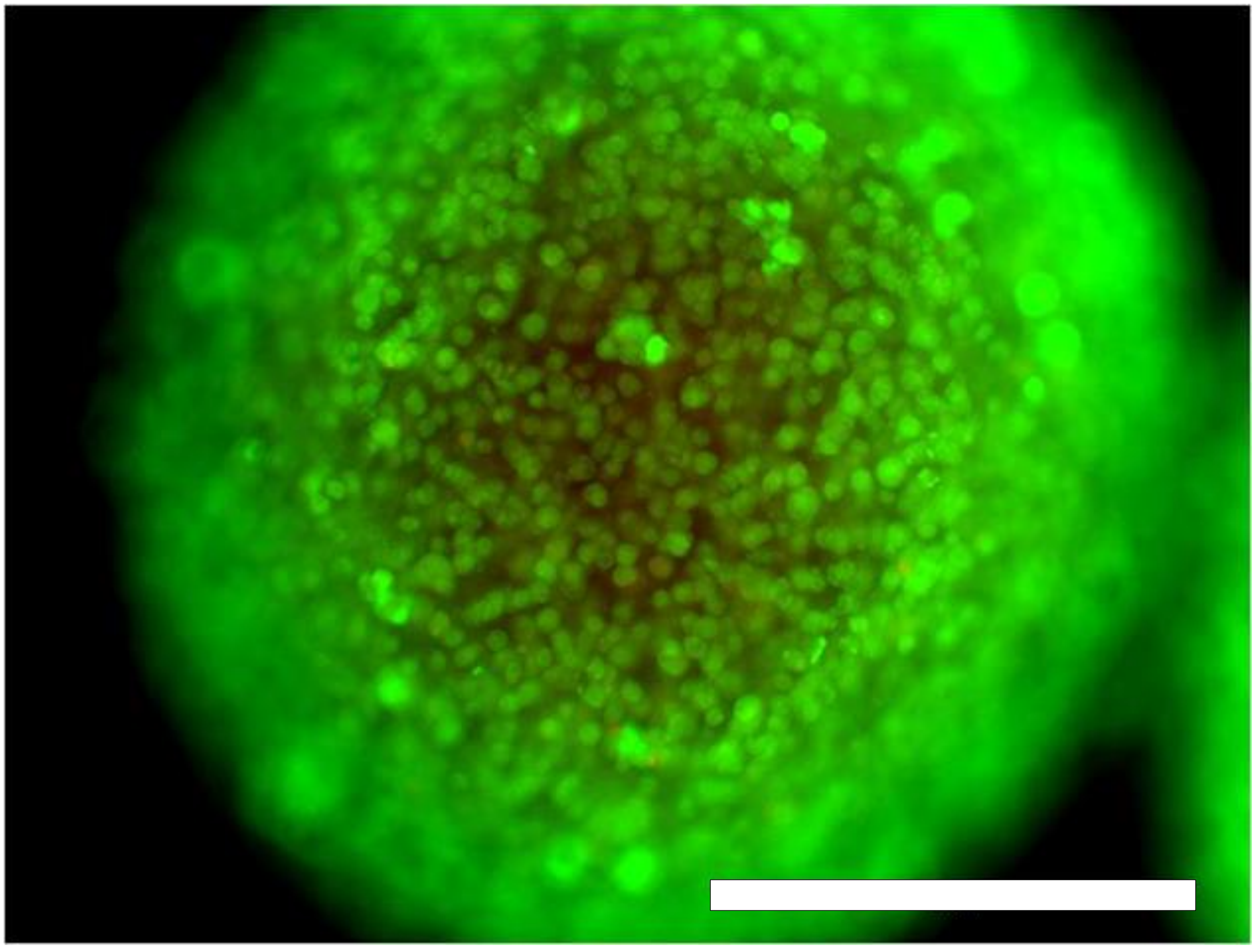

Supplementary Figure S2. Acridine orange/propidium iodide staining of alginate-encapsulated primary rat hepatocytes on Day 31 of culture. A 10x AMG objective was used with an AMG Evos FL inverted scope to capture the image. Image scale bar is 400 µm.

3 References

Chandrasekaran, P., Seagle, C., Rice, L., Macdonald, J., and Gerber, D.A. (2006). Functional analysis of encapsulated hepatic progenitor cells. *Tissue Eng* 12**,** 2001-2008.

Lecluyse, E.L., and Alexandre, E. (2010). Isolation and culture of primary hepatocytes from resected human liver tissue. *Methods Mol Biol* 640**,** 57-82.
